# Improved sex-specific cardiovascular risk prediction with multi-omics data in people with type 2 diabetes

**DOI:** 10.1186/s12933-025-03036-5

**Published:** 2025-12-24

**Authors:** Ruijie Xie, Christian Herder, Sha Sha, Hermann Brenner, Sigrid Carlsson, Ben Schöttker

**Affiliations:** 1 Division of Clinical Epidemiology of Early Cancer Detection, German Cancer Research Center, Im Neuenheimer Feld 581, 69120 Heidelberg, Germany; 2 Faculty of Medicine, Heidelberg University, 69115 Heidelberg, Germany; 3 Institute for Clinical Diabetology, German Diabetes Center (DDZ), Leibniz Center for Diabetes Research at Heinrich Heine University Düsseldorf, Düsseldorf, Germany; 4 German Center for Diabetes Research (DZD), Partner Düsseldorf, Munich-Neuherberg, Germany; 5 Department of Endocrinology and Diabetology, Medical Faculty and University Hospital Düsseldorf, Heinrich Heine University Düsseldorf, Düsseldorf, Germany; 6 Network Aging Research, Heidelberg University, Heidelberg, Germany; 7 Division of Urological Cancers, Department of Translational Medicine, Medical Faculty, Lund University, Lund, Sweden

**Keywords:** Proteomics, Multi-omics, Cardiovascular risk, Type 2 diabetes, Sex-specific, SCORE2-diabetes

## Abstract

**Background:**

To evaluate whether integrating proteomics, metabolomics, and a cardiovascular disease specific polygenic risk score (CVD-PRS) in the SCORE2-Diabetes model improves sex-specific 10-year prediction of major adverse cardiovascular events (MACE) in individuals with type 2 diabetes (T2D).

**Methods:**

Genome-wide association study (GWAS), plasma proteomics (with the Olink Explore 3072 platform), and metabolomics (with nuclear magnetic resonance spectroscopy by Nightingale Health) data were measured in the UK Biobank. A novel sex-specific protein algorithm was developed using bootstrap-LASSO (Least absolute shrinkage and selection operator) regression. The CVD-PRS and sex-specific metabolite algorithms were used from previous UK Biobank projects. In a subset of 990 participants with T2D, age 40–69 years, with no prior MACE, and complete multi-omics data, we evaluated, which omics data improved the SCORE2-Diabetes model performance using Harrell’s C-index.

**Results:**

Overall 9 proteins were selected for males and 7 for females and adding them to the SCORE2-Diabetes model significantly improved discrimination in the total population (C-index increase from 0.766 to 0.835 (*P* < 0.001)). Further adding of metabolites significantly improved model performance (C-index, 0.846, *P =* 0.035), which was mostly attributable to model improvement among males (∆C-index, 0.012, *P* = 0.078) but not among females (∆C-index, 0.004, *P* = 0.723). Further adding the CVD-PRS did not statistically significantly improve the SCORE2-Diabetes + proteomics + metabolomics model further in the total population (C-index, 0.848 (*P =* 0.070)).

**Conclusions:**

Sex-specific proteomic signatures markedly improved 10-year MACE risk prediction in individuals with T2D. In men but not in women, further integration of metabolomics may enhance model performance whereas adding the CVD-PRS is not needed. External validation is warranted.

**Graphical abstract:**

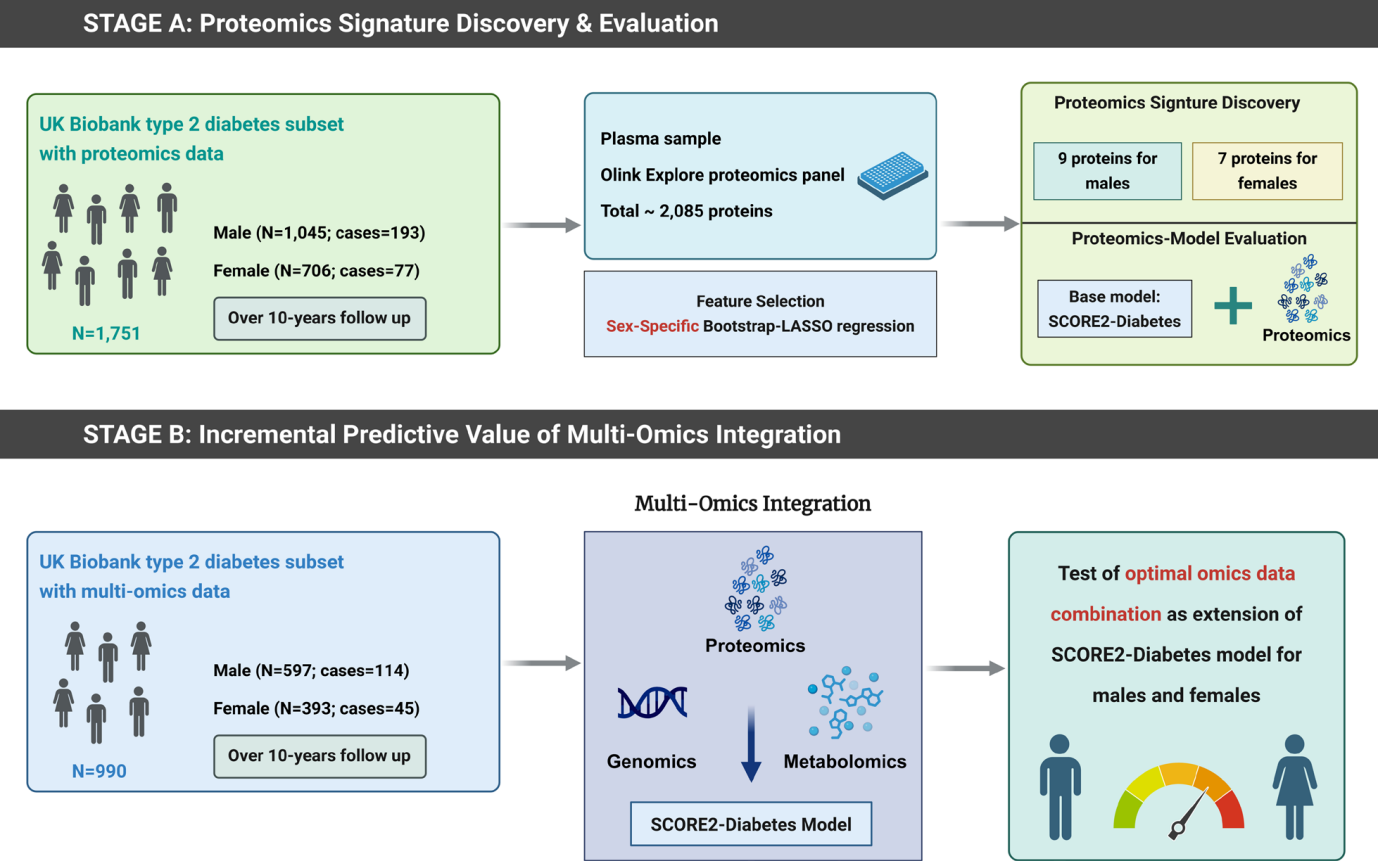

**Supplementary Information:**

The online version contains supplementary material available at 10.1186/s12933-025-03036-5.

## Introduction

Cardiovascular disease (CVD) remains the leading global cause of morbidity and mortality, and individuals with type 2 diabetes (T2D) are at substantially higher risk than the general population [1]. Despite advances in cardiovascular care, individuals with T2D continue to experience accelerated atherosclerosis and an elevated risk of major adverse cardiovascular events (MACE), underscoring the need for more precise and individualized risk stratification strategies [2, 3]. Additionally, sex-specific differences in cardiovascular outcomes have been increasingly recognized in individuals with T2D [4]. While men generally show higher absolute CVD risk at younger ages, women with T2D face a disproportionately greater relative increase in cardiovascular risk [5, 6].

In response to the need for more personalized prediction tools, the European Society of Cardiology recently introduced SCORE2-Diabetes, which is a sex-specific algorithm developed from large European cohort data to estimate the 10-year risk of MACE [7]. While this represents a significant improvement over previous general-population models, SCORE2-Diabetes primarily incorporates traditional clinical risk factors and does not sufficiently account for subclinical pathophysiological mechanisms or sex-specific variability, potentially limiting its predictive accuracy and clinical applicability [8, 9].

Omics-based methodologies, encompassing proteomics, metabolomics, and genomics, provide opportunities to improve cardiovascular risk prediction by quantifying disease-associated molecular signatures and uncovering underlying biological pathways not captured by traditional clinical assessments alone [10]. In particular, proteomics enables the identification of circulating proteins that reflect early pathophysiological changes and offers insights into the complex interplay of genetic, environmental, and metabolic factors contributing to CVD, especially in individuals with T2D [11, 12]. However, the application of proteomic biomarkers in cohorts of patients with T2D remains limited, and most previous studies have treated sex as a covariate rather than examining sex-specific biological differences directly [13, 14]. Given accumulating evidence of pronounced sex differences in proteomic signatures and cardiovascular pathogenesis, there is a critical need to develop and validate sex-specific proteomic risk models tailored to patients with T2D [15, 16].

The recent availability of large-scale proteomic data in the UK Biobank (UKB) provides an unprecedented opportunity to identify circulating protein biomarkers that improve cardiovascular risk prediction in individuals with T2D [17]. In this study, we first aimed to develop sex-specific proteomic signatures that enhance the predictive performance of the SCORE2-Diabetes model for 10-year MACE risk. We then evaluated whether the addition of metabolomic biomarkers and a cardiovascular polygenic risk score (PRS) could further improve prediction. The metabolomic biomarkers were derived from our previous study using the UKB, which demonstrated that 7 sex-specific metabolites significantly enhanced the predictive accuracy of the SCORE2-Diabetes model [18]. The CVD-PRS was provided by the UKB based on external GWAS and internal optimization [19]. Building on these multi-omics components, we investigated whether the integration of multiple omics layers could further refine sex-specific cardiovascular risk models in people with T2D.

## Methods

### Study population

The UKB is a large, population-based prospective cohort comprising 502,414 participants aged 40–69 years at recruitment (with few outliers), conducted between 13 March 2006 and 1 October 2010 across 22 assessment centers in England, Scotland, and Wales [20]. The cohort is predominantly White (approximately 94–95% White overall, and about 80% White British). The cohort includes comprehensive phenotypic and genotypic data, covering biomarkers, imaging, lifestyle, anthropometric, and genomic information.

The present study included two analytical cohorts (Supplemental Fig. S1). For the proteomics-based analysis, we excluded participants without proteomics data (*n* = 448,195), with more than 50% missing proteomics data (*n* = 1871), and individuals non-randomly selected for proteomics measurements who did not experience a MACE event during follow-up (*n* = 5282). Among the remaining 47,066 participants with usable proteomics data, we excluded individuals without diabetes (*n* = 44,365), with missing diabetes status (*n* = 189), diagnosed with type 1 diabetes (*n* = 488), with a history of MACE or missing outcome data (*n* = 254), or aged outside the SCORE2-Diabetes target range (< 40 or > 69 years; *n* = 19). Ultimately, 1751 participants with T2D were included in the proteomics-based analysis dataset. The study design is a case-cohort design with a randomly selected T2D sub-cohort with *n* = 1672 study participants (of whom *n* = 191 developed MACE during follow-up) and *n* = 79 additional MACE cases [21].

The multi-omics-based analysis used the same exclusion criteria but additionally excluded participants with missing metabolomics data (*n* = 750) or PRS data (*n* = 11), resulting in 990 participants with T2D and complete multi-omics.

### Base model: SCORE2-diabetes

The SCORE2-Diabetes is a developed and validated sex-specific cardiovascular risk model tailored for adults with T2D aged 40–69 years [7]. The model incorporates the following variables: age, sex, systolic blood pressure (SBP), current smoking status, age at diabetes diagnosis, high-density lipoprotein cholesterol (HDL-C), total cholesterol (TC), glycated hemoglobin (HbA_1c_), and estimated glomerular filtration rate (eGFR). Age, sex, and smoking status were self-reported through standardized touchscreen questionnaires at baseline. SBP was measured in the seated position using an automated digital sphygmomanometer (Omron HEM-7015IT) on the left arm, with the mean of two readings used for analysis. HDL-C and total cholesterol concentrations were determined from non-fasting blood samples using enzymatic colorimetric assays on the Beckman Coulter AU5800 platform. HbA_1c_ was measured via high-performance liquid chromatography (Bio-Rad Variant II Turbo). eGFR was calculated using the CKD-EPI 2009 creatinine equation.

### Proteomics data

Proteomic profiling was conducted on EDTA-plasma samples collected at baseline. The assay protocols, including sample handling and selection procedures, have been described previously [22]. In brief, a total of 2923 unique proteins were measured using the Olink Explore 3072 platform (Olink Proteomics, Uppsala, Sweden). Olink utilizes the Proximity Extension Assay (PEA) method that targets proteins via pairs of antibodies linked to complementary oligonucleotides [23]. As outlined in our prior study [24], proteins with more than 20% missing values or with over 25% of values below the detection limit were excluded (*n* = 838). Ultimately, 2085 proteins from the Olink Explore platform were included in the proteomic biomarker selection process. In this study, sex-specific biomarker selection was performed using least absolute shrinkage and selection operator (LASSO) with bootstrap resampling to identify the most predictive proteins for cardiovascular risk among individuals with T2D. This bootstrap resampling approach serves as a form of internal validation for the feature selection process, aiming to ensure the stability of the selected protein signature and to mitigate the risk of overfitting.

### Metabolomic data

Plasma metabolite profiling was performed by Nightingale Health using a targeted nuclear magnetic resonance (NMR) spectroscopy platform [25]. This approach captured a broad spectrum of 249 circulating metabolites, including lipid subclasses, fatty acids, amino acids, and other low-molecular-weight compounds. Of these, 168 were quantified as absolute concentrations, encompassing 61 composite biomarkers derived from 107 directly measured metabolites, while the remaining 81 were reported as ratio measures. In our previous study [18], we applied sex-specific LASSO regression to the 249 metabolites and identified a parsimonious signature comprising 7 metabolites (3 common to both sexes, 3 male-specific and 1 female-specific; Supplemental Table S1). Incorporation of these metabolites into the SCORE2-Diabetes model increased C-index from 0.660 to 0.678 in the total population (*P* = 0.037), with ΔC-index = + 0.025 in men (from 0.632 to 0.657) and ΔC-index = + 0.009 in women (from 0.670 to 0.679). In the present analysis, we evaluated only these 7 pre-selected metabolites.

### Polygenic risk score for CVD

The PRS for CVD was obtained from the UKB PRS Release [15]. This pre-computed score incorporates 1.42 million genetic variants with non-zero weights. The score was developed using a Bayesian modeling framework, initially trained on meta-analyzed summary statistics from nine external GWAS datasets. These summary statistics were sourced from large-scale international consortia, primarily CARDIoGRAMplusC4D and MEGASTROKE, where genotyping was conducted on a variety of Illumina and Affymetrix arrays, with Illumina platforms being predominant [26]. Subsequently, it was optimized using individual-level data from the UK Biobank cohort, for which genotyping was conducted using the UK Biobank Axiom and UK BiLEVE Axiom arrays [15]. According to internal validation analyses, this CVD-PRS demonstrates robust predictive performance and is superior to previously published scores [15, 27].

### Outcome ascertainment

The primary outcome was the occurrence of a MACE, defined in accordance with the SCORE2-Diabetes model as a composite of cardiovascular death, non-fatal myocardial infarction, or non-fatal stroke [7]. Non-fatal events were identified through linked primary care records and hospital inpatient data obtained via the Hospital Episode Statistics system. Information on dates and causes of death was obtained from national death registries, specifically the National Health Service (NHS) Information Centre for England and Wales and the NHS Central Register for Scotland. Participants were followed from baseline until the earliest of the following: occurrence of a MACE event, death from any cause, or completion of the 10-year follow-up period. A list of diagnostic codes and outcome definitions is provided in Supplemental Table S2.

### Statistical analyses

#### General remarks

All analyses were performed using R software (version 4.4.0, R Foundation for Statistical Computing, Vienna, Austria). Statistical significance was defined as *P*-values < 0.05 for two-sided tests. Missing data in SCORE2-Diabetes variables (with HDL-C having the highest missing rate of 12.0%) and multi-omics measurements (mostly complete, with up to 20% missing for a few proteins) were single imputed using the chained equations method with random forest algorithms implemented in the R package *missForest* (version 1.5) [26]. The missing values per variable are shown in the legend of Table 1. To the best of our knowledge, all missing values were missing at random. The variable distributions did not change by missing value imputation. In sensitivity analysis, multiple imputation of 5 data sets led to almost identical associations as MissForest imputation (data not shown). All Cox proportional hazards models accounted for the case-cohort design using inverse-probability Barlow weights [27].

**Table 1:** Baseline characteristics of participants included in the proteomics and multi-omics analyses from the UK biobank

Baseline characteristics	Proteomics cohort (*N* = 1751)	Multi-omics cohort (*N* = 990)	*P*-value
Male sex, N (%)	1045 (59.7%)	597 (60.3%)	0.796
Age (years)	60.3 (6.6)	60.5 (6.6)	0.364
Current smoker, N (%)	187 (10.7%)*	103 (10.4%)^#^	0.879
Systolic blood pressure (mmHg)	144.5 (17.7)*	144.3 (17.6)^#^	0.757
Total cholesterol (mmol/L)	4.5 (1.0)*	4.5 (1.0)^#^	0.790
HDL cholesterol (mmol/L)	1.2 (0.3)*	1.2 (0.3)^#^	0.671
Age at diabetes diagnosis (years)	55.0 (6.5)*	55.2 (6.4)^#^	0.335
HbA_1c_ (mmol/mol)	51.8 (12.9)*	51.7 (12.4)^#^	0.833
eGFR (ml/min/1.73m^2^)^a^	88.3 (16.1)*	87.4 (16.4)^#^	0.176
Lipid-lowering medication, N (%)	1308 (74.7%)	750 (75.8%)	0.556
Anti-hypertensive medication, N (%)	1087 (62.1%)	632 (63.9%)	0.374

Abbreviations: eGFR, estimated Glomerular Filtration Rate; HbA_1c_, glycated hemoglobin; HDL, high-density lipoprotein

^a^The eGFR was calculated with the CKD-EPI 2009 equation

Values are expressed as mean (standard deviation) for continuous variables, and as N (%) for categorical variables. *P*-values were calculated using t-tests for continuous variables and χ^2^ tests for categorical variables

*In the proteomics cohort (*N* = 1751), the following variables contained missing data before imputation: HDL cholesterol (210 missing, 12.0%; data available for 1541 participants), eGFR (105 missing, 6.0%; data available for 1646 participants), HbA_1_c (120 missing, 6.9%; data available for 1631 participants), total cholesterol (101 missing, 5.8%; data available for 1650 participants), age at diabetes diagnosis (115 missing, 6.6%; data available for 1636 participants), current smoking status (14 missing, 0.8%; *n* = 1737), and systolic blood pressure (5 missing, 0.3%; data available for 1746 participants). All other baseline variables were complete

#In the multiomics cohort (*N* = 990), missingness was similar: HDL cholesterol (118 missing, 11.9%; *n* = 872), eGFR (62 missing, 6.3%; *n* = 928), HbA_1c_ (67 missing, 6.8%; *n* = 923), total cholesterol (56 missing, 5.7%; *n* = 934), age at diabetes diagnosis (63 missing, 6.4%; *n* = 927), systolic blood pressure (3 missing, 0.3%; *n* = 987), and current smoking status (8 missing, 0.8%; *n* = 982). All other baseline variables were complete

#### Evaluation of model performance and incremental value of multi-omics integration

Model development and evaluation followed a two-stage approach. In the first stage, proteomics-based models were derived and evaluated in models adjusted for the variables of the SCORE2-Diabetes model using the subset of T2D patients with available proteomics data (*N* = 1751) for best statistical power. To obtain stable and parsimonious protein signatures, we applied a bootstrap-LASSO stability-selection protocol. Specifically, for each sex, we generated 1000 bootstrap resamples and fitted a LASSO-penalized Cox model in each resample, with the regularization parameter (λ) determined by ten-fold cross-validation selecting the value that minimized the cross-validated error (λ_min). Proteins selected in at least 95% of the bootstrap iterations were retained to ensure robustness against stochastic variation. The retained proteins were then refit in unpenalized Cox models adjusted for all SCORE2-Diabetes variables to obtain final coefficients and model performance.

In the second stage, the additional predictive value of signatures derived from genomic, metabolomic, and proteomic data (Supplemental Table S1), when added to the SCORE2-Diabetes model, was assessed in the subset of T2D patients with complete multi-omics data (*N* = 990). These three multi-omics layers were added sequentially to the SCORE2-Diabetes model, and model performance was evaluated. Finally, the best-performing omics layers were added one by one to the SCORE2-Diabetes model until the addition of another layer did not result in a statistically significant improvement in model discrimination.

Model discrimination was evaluated using Harrell’s C-index and statistical significance for differences between correlated C-indices was assessed using the method proposed by Kang et al., implemented in the R package *compareC* (version 1.3.2) [28]. Moreover, the incremental C-index for each individual biomarker was estimated to assess its independent contribution to model discrimination.

Additionally, risk reclassification was quantified using the net reclassification index (NRI) and integrated discrimination index (IDI) [29]. Predefined cardiovascular risk categories (0–15%, > 15–30%, and > 30%) were used to calculate the NRI, comparing the proportion of correctly reclassified individuals against the SCORE2-Diabetes model [7]. Model calibration was assessed by plotting observed versus predicted MACE event rates across deciles of predicted absolute risk.

#### Associations of selected multi-omics biomarkers with MACE

To quantify the association between individual biomarkers and 10-year MACE risk, each selected biomarker was entered separately into a Cox proportional hazards model, adjusted for the SCORE2-Diabetes variables. Hazard ratios (HRs) and 95% confidence intervals (CIs) per one standard deviation increment were reported, stratified by sex. Models were fitted using the *survival* package (version 3.5-5) in R.

#### Correlation matrix

To assess redundancy among selected biomarkers, Spearman correlation coefficients were calculated for the selected proteins, metabolites, and the CVD-PRS in males and females separately.

## Results

### Baseline characteristics and MACE incidence

Table 1 summarizes the baseline characteristics of the 1751 UKB participants with T2D included in the proteomics-based analysis cohort and the 990 participants in the multi-omics cohort. In the proteomics cohort, the mean age was 60.3 ± 6.6 years and 59.7% of participants were male. The multi-omics cohort was similar, with a mean age of 60.5 ± 6.6 years and 60.3% male participants. The distributions of smoking status, SBP, TC, HDL-C, age at diabetes diagnosis, HbA_1c_, eGFR, use of lipid-lowering and anti-hypertensive medication were also comparable between the two cohorts, with all *P*-values > 0.05. During the 10-year follow-up, 270 participants (15.4%) in the proteomics cohort experienced a MACE, including 193 males and 77 females. In the multi-omics cohort, 159 participants (16.1%) developed MACE, among whom 114 were males and 45 females.

### Predictive performance of proteomics extended SCORE2-Diabetes model

Overall, 15 proteomic biomarkers were selected by LASSO regression for their ability to enhance the predictive performance of the SCORE2-Diabetes model: 8 were male-specific, 6 were female-specific, and one, WFDC2 (WAP four-disulfide core domain protein 2), was common to both sexes (see receiver operating characteristic (ROC) curves in Supplemental Fig. S2). Supplemental Table S3 presents the β-coefficients of the proteomics-extended SCORE2-Diabetes model. As shown in Table 2, the inclusion of these proteins markedly improved model discrimination. The C-index increased from 0.725 to 0.810 (ΔC-index, 0.085; *P* < 0.001) in the total population, with similarly large improvements in males (from 0.700 to 0.797; ΔC-index, 0.097; *P* < 0.001) and females (from 0.740 to 0.835; ΔC-index, 0.095; *P* < 0.001). This gain in discrimination was corroborated by a substantial improvement in risk reclassification, highlighted by a categorical NRI of 24.2%, continuous NRI of 31.6%, and a significant increase in the IDI of 0.098 for the overall cohort. The calibration curve illustrated good agreement between observed and predicted risks, highlighting the reliable performance of the proteomics extended SCORE2-Diabetes model (Supplemental Fig. S3).

**Table 2:** Metrics of the predictive performance of the SCORE2-Diabetes model for 10-year MACE risk without and with extension by proteins (*N* = 1751)

Metrics	Male^a^	Female^b^	Overall
C-Statistics (SCORE2-Diabetes)	0.700 (0.659, 0.742)	0.740 (0.682, 0.799)	0.725 (0.697, 0.757)
C-Statistics (+ Proteins)	0.797 (0.760, 0.833)	0.835 (0.789, 0.881)	0.810 (0.782, 0.837)
*P*-values for C-Statistics comparisons	**< 0.001**	**< 0.001**	**< 0.001**
Categorical NRI total (%)	**51.2 (18.3**,** 67.9)**	**29.5 (18.4**,** 42.1)**	**24.2 (15.9**,** 33.1)**
Categorical NRI events (%)	− 12.6 (− 17.1, 6.8)	**34.9 (24.5**,** 47.1)**	19.1 (− 2.1, 37.6)
Categorical NRI non-events (%)	**63.9 (18.6**,** 78.1)**	− **5.4 (**− **7.6**, − **2.4)**	5.1 (− 1.6, 28.3)
Continuous NRI (%)	**32.3 (27.4**,** 39.6)**	**36.8 (25.1**,** 47.1)**	**31.6 (25.6**,** 38.5)**
IDI	**0.105 (0.058**,** 0.146)**	**0.111 (0.028**,** 0.210)**	**0.098 (0.052**,** 0.130)**

Boldvalues indicate statistically significant differences between models (P < 0.05)

Abbreviations: IDI, integrated discrimination index; MACE, major cardiovascular events; NRI, net reclassification index

^a^Proteins included in the combined model for males were: ACTA2, CHGA, CRNN, ENPP5, GAST, NT-proBNP, POSTN, TRIM21, WFDC2

^b^Proteins included in the combined model for females were: CTRC, EFHD1, HS3ST3B1, IL22, SMOC1, TNC, WFDC2

### Predictive performance of multi-omics extended SCORE2-Diabetes model

**Fig. 1:**
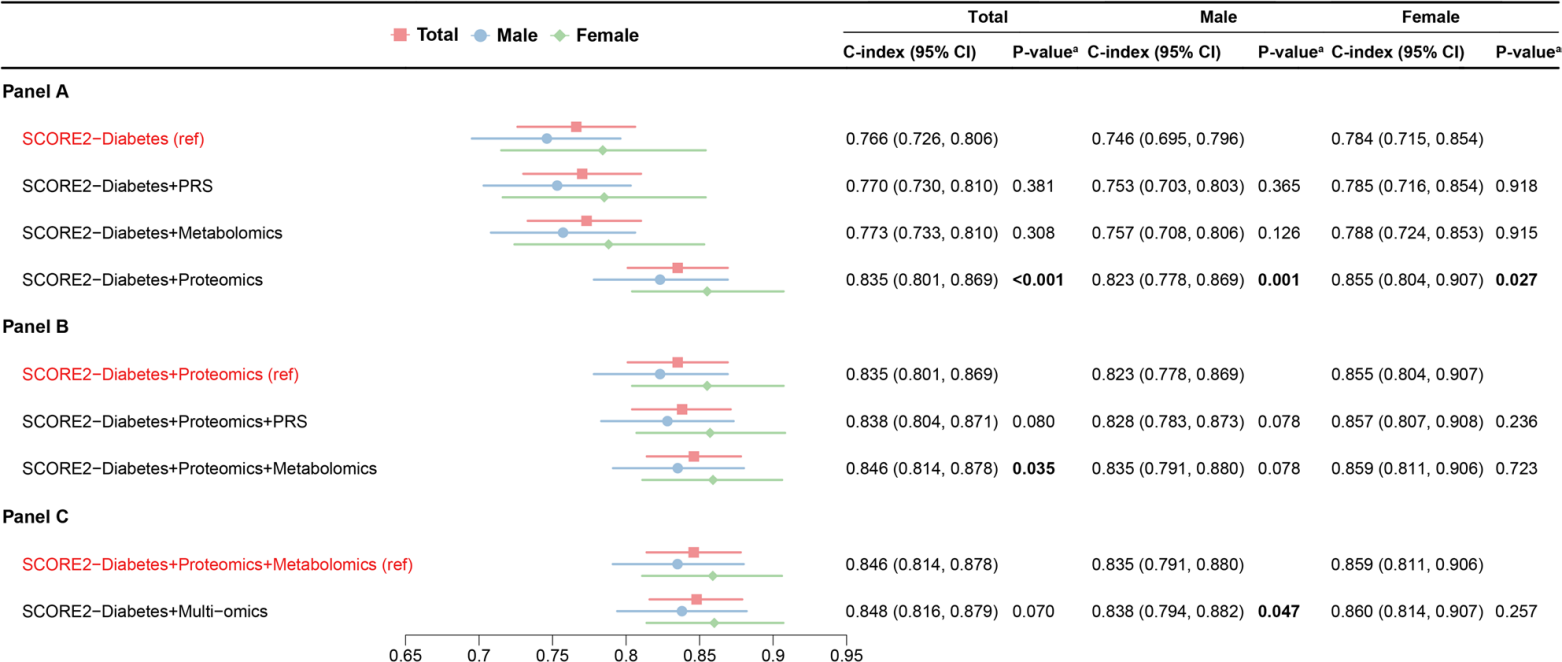
Discrimination performance of single-omics and multi-omics extensions to the SCORE2-Diabetes in 10-year MACE risk prediction (*N* = 990). ^a^*P*-values indicate statistical comparisons of C-indices against the corresponding reference model Panel **A** shows the C-index with 95% confidence intervals for models extending SCORE2-Diabetes with individual omics layers (PRS, metabolomics, or proteomics). Panel **B** compares multi-omics combinations using SCORE2-Diabetes + proteomics as the reference. Panel **C** contrasts the full multi-omics model with the SCORE2-Diabetes + proteomics + metabolomics model as the reference. CI, confidence interval; PRS, polygenic risk score; MACE, major adverse cardiovascular event

Figure 1 evaluates which combination of omics data yielded the highest C-index for 10-year MACE prediction. Supplemental Table S5 adds the reclassification statistics NRI and IDI for these model comparisons. Among all single-omics extensions to the SCORE2-Diabetes model, proteomics led to the largest improvement in the total population (C-index: 0.835 vs. 0.766; ΔC-index, 0.069; *P* < 0.001), in males (0.823 vs. 0.746; ΔC-index, 0.077; *P* = 0.001), and in females (0.855 vs. 0.784; ΔC-index, 0.071; *P* = 0.027), and was therefore selected as the reference model for subsequent comparisons. Using the proteomics-extended SCORE2-Diabetes model as the reference, the addition of the CVD-PRS did not significantly improve model performance in the total population, in males, or in females. The inclusion of metabolomic biomarkers led to a significant increase in the C-index in the total population (C-index: 0.846 vs. 0.835; ΔC-index, 0.011; *P* = 0.035; NRI: − 0.8%, *P* = 0.811; IDI: 0.001, *P* = 0.812), which was highly attributable to the model improvement among males, which almost reached statistical significance in C-index increase and NRI but not IDI statistics (C-index: 0.835 vs. 0.823; ΔC-index, 0.012; *P* = 0.078; NRI: 9.8%, *P* = 0.086; IDI: − 0.005, *P* = 0.792). Further incorporation of the CVD-PRS into the model combining SCORE2-Diabetes variables, proteomics, and metabolomics did not significantly enhance predictive performance in the total population or females. Although a marginal increase in the C-index was statistically significant in males, the reclassification statistics did not indicate a model improvement (ΔC-index, 0.003; *P* = 0.047; NRI: − 2.6%, *P* = 0.220; IDI: − 0.002, *P* = 0.495). Supplemental Table S5 presents the β-coefficients for the variables included in the sex specific, multi-omics extended SCORE2-Diabetes models. Supplemental Fig. S4 shows the ROC curves and Supplemental Fig. S5 the model calibration curves of the SCORE2-Diabetes with and without extension by multi-omics data.

### Incremental contributions of individual biomarkers to improved model performance

**Fig. 2:**
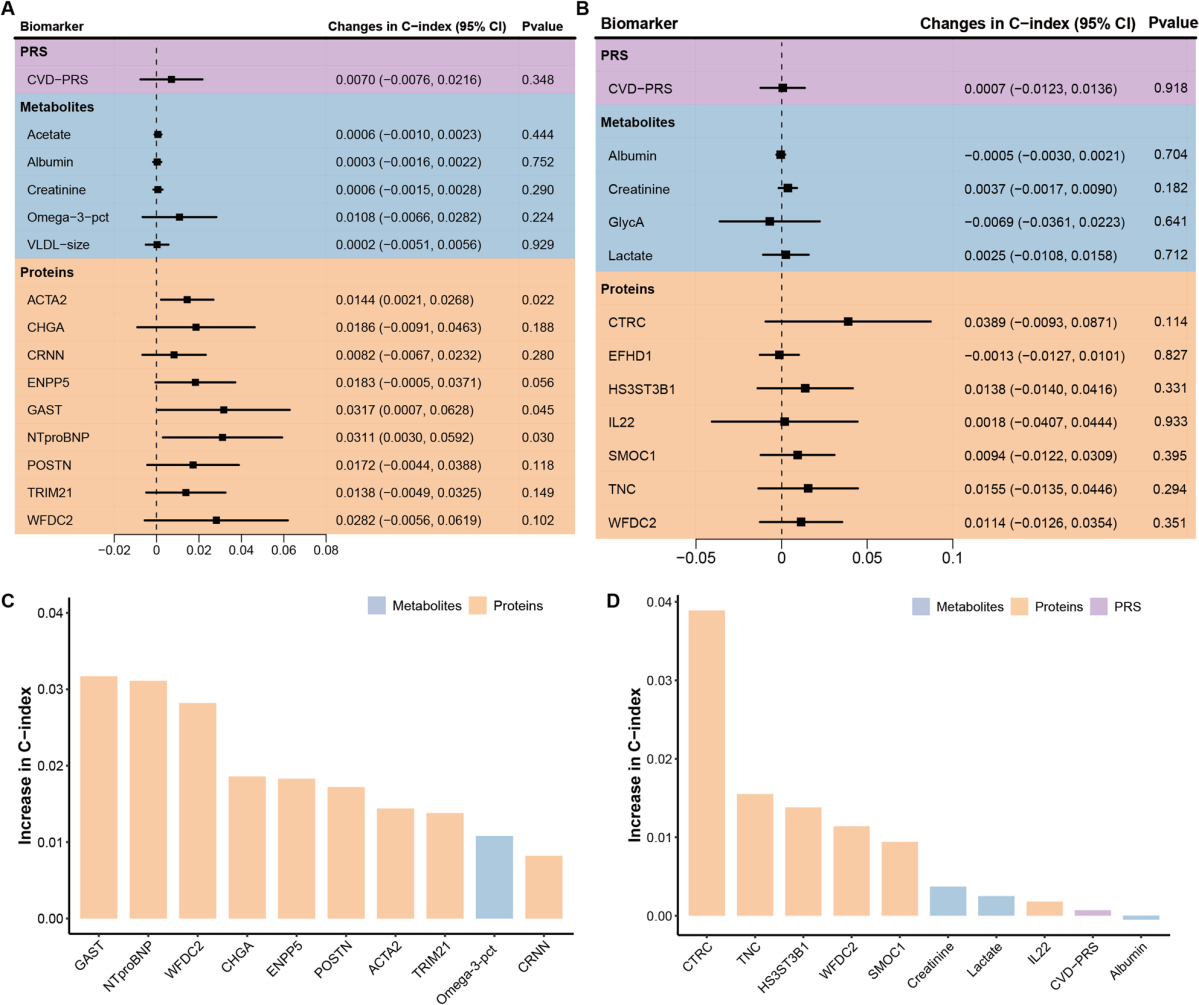
Incremental predictive value of selected multi-omic biomarkers added to the SCORE2-Diabetes model for 10-year MACE risk prediction (*N* = 990). Panel **A** and **B** show the changes in C-index when each biomarker is individually added to the SCORE2-Diabetes model in male and female participants. Panel **C** and **D** illustrate the top 10 biomarkers ranked by individual C-index gains in males and females. ACTA2, Actin Alpha 2; CHGA, Chromogranin-A; CRNN, Cornulin; CTRC, Chymotrypsin-C; CVD, cardiovascular disease; EFHD1, EF-hand domain-containing protein D1; ENPP5, Ectonucleotide pyrophosphatase/phosphodiesterase family member 5; GAST, Gastrin; HS3ST3B1, Heparan sulfate glucosamine 3-O-sulfotransferase 3B1; IDI, integrated discrimination improvement; IL22, Interleukin-22; MACE, major adverse cardiovascular event; NRI, net reclassification improvement; NT-proBNP, N-terminal prohormone of brain natriuretic peptide; Omega-3-pct, The Omega-3 fatty acids percentage of total fatty acids; PRS, polygenic risk score; SMOC1, SPARC-related modular calcium-binding protein 1; TNC, Tenascin-C; TRIM21, E3 ubiquitin-protein ligase TRIM21; VLDL-size, Average diameter for very-low-density lipoprotein particles; WFDC2, WAP four-disulfide core domain protein

Figure 2A and B present the increase in C-index of each individual selected multi-omics biomarker when added to the SCORE2-Diabetes model for males and females, respectively. In males, GAST (Gastrin) and NT-proBNP (N-Terminal Pro-Brain Natriuretic Peptide) conferred the largest individual increase in the C-index (ΔC-index = + 0.0317 and + 0.0311, respectively). In females, CTRC (Chymotrypsin-C) was the most influential biomarker (ΔC-index = + 0.0389). In males, 9 out of the 10 most important contributors were proteins (Fig. 2C), while in females, it was 6 out of 10 (Fig. 2D). However, the top 5 predictors among females were all proteins, which further underscores the higher predictive ability of proteomic biomarkers compared to metabolomic and genomic markers for both sexes.

### Associations of selected biomarkers with MACE incidence

Supplemental Figure S6 shows hazard ratios for the associations between the selected multi-omics biomarkers and 10-year MACE risk in male and female participants, respectively. In both sexes, all selected proteins except one (CRNN (Cornulin) in males) were significantly associated with MACE. In contrast, all selected metabolites except one (omega-3-pct (Omega-3 fatty acids percentage of total fatty acids) in males) were not significantly associated with MACE in both sexes. The CVD-PRS was only significantly associated with MACE among males.

### Correlations among selected biomarkers

Supplemental Figure S7 displays the Spearman correlation matrices for the selected proteins, metabolites, and the CVD-PRS in males and females, respectively. Correlations between biomarkers across omics layers were weak overall. No correlation coefficient exceeded 0.1 between the CVD-PRS and any selected protein or metabolite in either sex. With one exception (CHGA (Chromogranin-A) and GAST among males (*r* = 0.64)), no pairwise correlation exceeded *r* = 0.47.

## Discussion

### Summary of the findings

In this study of individuals with T2D from the UKB, we developed sex-specific risk models that integrated proteomic biomarkers into the established SCORE2-Diabetes algorithm to improve 10-year prediction of MACE. A total of 15 proteins were selected using a bootstrap-LASSO approach, including one protein (WFDC2) shared across sexes, 8 specific to males, and 6 specific to females. The inclusion of these proteomic signatures markedly enhanced model discrimination for both sexes. In the total population, an additional gain in model performance was observed by further adding metabolites, which was attributable to model improvement among males but not among females. Further adding the CVD-PRS slightly improved the model’s C-index but not reclassification statistics among men but not women. This sex difference was supported by a lack of statistically significant associations of all selected metabolites and the CVD-PRS with MACE incidence in females, whereas the CVD-PRS and one metabolite showed statistically significant associations with MACE incidence in males.

### Comparison with previous studies

To our knowledge, this is the first study to develop sex-specific proteomic signatures for cardiovascular risk prediction in individuals with T2D. Most previous studies with sex-specific analyses have investigated proteomic biomarkers in general populations with limited attention to individuals with T2D [30–33]. These studies often overlooked the distinct cardiovascular risk profiles and pathophysiological mechanisms unique to populations with diabetes. In our prior analysis using the UKB cohort without diabetes (*N* = 47,382), we applied the same sex-specific biomarker selection framework and identified 18 proteins associated with MACE [34]. Interestingly, only two proteins overlapped with the current diabetes-specific results (NT-proBNP and WFDC2 in males; WFDC2 alone in females). This limited overlap in selected proteins underscores the need to develop proteomic risk models tailored to individuals with T2D.

Prior proteomics-based cardiovascular risk prediction studies in populations with diabetes have utilized less extensive protein panels without sex-specific biomarker selection [13, 14, 35–38]. For example, the SUMMIT study employed 6 plasma proteins across five European T2D cohorts (*N* = 2310) and observed a notable improvement in AUC from 0.59 to 0.72 for 5-year cardiovascular risk prediction [13]. Similarly, Nowak et al. utilized 20 proteins measured via the Olink platform in pooled Swedish T2D cohorts (*N* = 1211), and achieved a C-index increase from 0.686 to 0.747 [35]. The EXAMINE trial (*N* = 5131), which focused on T2D patients with recent myocardial infarction, demonstrated that adding a protein panel including NT-proBNP and Galectin-9 improved the C-index by over 0.05 and significantly enhanced reclassification metrics [36]. Although these studies support the utility of proteomics in T2D populations, their lack of sex-specific biomarker selection potentially limits their predictive precision.

Our study specifically developed sex-specific proteomic signatures for cardiovascular risk prediction among individuals with T2D. Unlike previous studies that required extensive protein panels, we achieved comparable or superior predictive accuracy with a concise set of biomarkers (9 proteins for males and 7 for females). Our study was the first to test whether additional inclusion of metabolomics and genomic data could further improve MACE prediction in patients with T2D. Further studies are needed to corroborate our results.

### Potential biological mechanisms for the selected proteins in CVD development

The 15 proteins identified in this study are involved in a range of biological processes relevant to cardiovascular risk in individuals with T2D, including pathways related to cardiac stress, fibrosis, inflammation, and metabolic dysfunction. Several proteins, including NT-proBNP, CHGA, and EFHD1 (EF-hand domain-containing protein D1), are associated with cardiac stress and neurohumoral signaling [39–41]. NT-proBNP is a well-established biomarker of myocardial wall stress and subclinical heart dysfunction, extensively validated in populations with and without diabetes [42, 43]. CHGA is a prohormone co-released with catecholamines that influences autonomic regulation and vascular tone through active peptides such as catestatin [40]. EFHD1 is a mitochondrial calcium-binding protein linked to oxidative stress and mitochondrial dysfunction, which may contribute to cardiomyocyte injury in metabolically impaired states such as T2D [41].

Several other proteins are involved in fibrosis and extracellular matrix remodeling, processes known to drive plaque instability and adverse ventricular remodeling. WFDC2, the sole protein selected in both sexes in our study, is associated with myocardial and renal fibrosis, potentially via mechanisms involving collagen deposition regulation and protease inhibition [44, 45]. TNC (Tenascin-C), POSTN (Periostin), ACTA2 (Actin Alpha 2), and SMOC1 (SPARC-related modular calcium-binding protein 1) are matricellular proteins or contractile regulators that influence fibroblast activation, vascular smooth muscle cell proliferation, and extracellular matrix turnover [46–49]. These processes contribute to vascular stiffening, neointimal hyperplasia, and fibrotic remodeling, particularly relevant in T2D patients who experience accelerated atherosclerosis and impaired tissue repair [50].

Inflammatory and immune-regulatory mechanisms are also reflected among the selected proteins. IL22 (Interleukin-22) is a pleiotropic cytokine involved in mucosal immunity and tissue regeneration; however, in chronic inflammatory states such as T2D, it has been associated with endothelial dysfunction and atherogenesis [51]. TRIM21 (E3 ubiquitin-protein ligase TRIM21) functions as an intracellular Fc receptor and E3 ligase, regulating immune signaling and oxidative stress pathways associated with vascular inflammation [52]. CRNN is an epithelial stress-response protein associated with inflammation, potentially reflecting systemic inflammatory burden or metabolic stress relevant to cardiovascular risk in T2D [53].

Several proteins are involved in metabolic-vascular regulation and endothelial homeostasis. GAST is primarily known for its role in gastrointestinal function but has also been linked to hypertension and subclinical atherosclerosis, possibly through stimulation of vascular smooth muscle [54]. CTRC is a pancreatic serine protease that may reflect systemic proteolytic activity and metabolic disturbances in diabetes, and has recently been linked to coronary artery disease in a sex-specific manner [55, 56]. This may reflect a broader disruption of protease-antiprotease balance in T2D, potentially affecting endothelial integrity or vascular remodeling, although the precise mechanisms remain unclear. ENPP5 (Ectonucleotide pyrophosphatase/phosphodiesterase family member 5) is part of the ectonucleotide pyrophosphatase family and has been implicated in cellular senescence, vascular stiffening, and low-grade inflammation [57]. HS3ST3B1 (Heparan sulfate glucosamine 3-O-sulfotransferase 3B1) encodes a 3-O-sulfotransferase that modifies the fine structure of heparan sulfate chains [58]. By altering sulfation patterns on endothelial surfaces, it may indirectly modulate leukocyte adhesion and inflammatory signaling, as heparan sulfate structure is known to influence cytokine binding and vascular homeostasis. These mechanisms may underlie early metabolic-endothelial dysfunction in T2D and help explain the elevated cardiovascular risk observed in this population.

### Clinical translation and feasibility

Previous large-scale proteomics studies often selected a large number of proteins, significantly increasing measurement complexity, cost, and limiting clinical feasibility [59, 60]. Our approach substantially reduces this burden by utilizing concise, sex-specific protein panels (9 proteins for males and 7 for females). For example, it would be possible to design an Olink Focus panel, which allows simultaneous measurement of up to 21 proteins with absolute concentrations (https://olink.com/products/olink-focus). After relatively high investment costs once to develop such a panel, the scaling up of measuring it in clinical routine is feasible at low costs. The current costs are approx. €67 per sample but are expected to decrease if assays are translated from scientific use for a few samples to large-scale use in clinical routine. As our results need to be externally validated anyway before they can be translated in clinical routine, it would be ideal if further studies would use PEA or a comparable technology to measure the selected proteins with absolute concentrations.

Our results suggest that integrating further multi-omics layers may improve the predictive accuracy in males, but the clinical translation of these additional biomarker platforms is challenging with regard to costs and timely measurement, as additional laboratories would need to be involved [61]. Future studies should carefully weigh the incremental predictive gains (which were particularly low for the CVD-PRS) by additional omics biomarker measurements in men against the associated measurement costs and feasibility. In women, the marginal improvements in model accuracy did not reach statistical significance in our study, which suggests that a protein-extended SCORE2-Diabetes model is sufficient for MACE risk assessment in women with T2D.

### Strengths and limitations

This study’s strengths include the availability of multi-omics data from proteomics, metabolomics, and genomics in a large sample of people with T2D with 10-year MACE follow-up. The use of sex-specific modeling accounted for biological differences between males and females and represents an incremental step toward personalized cardiovascular disease prevention.

Several limitations must also be considered. First, a limitation is that the number of MACE cases was not sufficient for a separate internal validation using, for instance, a data split into dedicated training and testing sets or k-fold cross-validation. Consequently, the reported improvements in predictive performance may be overly optimistic, and replication in independent cohorts is necessary to confirm generalizability [62]. Second, the metabolomics data derived from the Nightingale Health platform were limited to approximately 250 biomarkers, and more comprehensive, untargeted metabolomics approaches based on mass spectrometry might yield superior predictive performance. Third, UK Biobank participants are almost entirely of European ancestry, with approximately 95% being White and around 80% being White British, and were aged 40–69 years at recruitment. As these volunteers are generally healthier than the broader population with diabetes, the direct extrapolation of our findings to non-European ethnic groups, older individuals, or higher risk secondary prevention cohorts is limited. Fourth, although beta coefficients are provided for the proteomics-extended and multi-omics data extended SCORE2-Diabetes model for external validation, recalibration may be necessary when these models are applied to populations with different baseline CVD risk or if alternative omics measurement platforms were used. Finally, an in-depth cost-benefit or cost-effectiveness analysis about implementing either the proteomics-extended or multi-omics data extended risk models into routine clinical practice was beyond the scope of this study but remains essential.

## Conclusion

In this study, we identified 9 proteins in men and 7 proteins in women that improved 10-year MACE prediction beyond the SCORE2-Diabetes model in individuals with T2D. Among the omics layers, proteomics provided the largest incremental gain in predictive accuracy. In women, adding metabolomics or a CVD-PRS did not further enhance the performance of the protein-extended model. In men, incorporating metabolomics showed additional model improvement, whereas further adding a CVD-PRS did only marginally improve its performance. External validation and cost-effectiveness analyses are now warranted before these improved SCORE2-Diabetes models can be recommended for clinical routine. We hypothesize that adding the measurement of the few selected proteins specific for males or females might be feasible and cost-effective in clinical practice. These concise, sex-specific biomarker panels would be a first step toward more precise cardiovascular risk stratification.

## Supplementary Information

Below is the link to the electronic supplementary material.

Supplementary Material 1

## Data Availability

Data from the UK Biobank are available to bona fide researchers upon application through the UK Biobank Access Management System (https://www.ukbiobank.ac.uk/enable-your-research/apply-for-access).

## Abbreviations

ACTA2: Actin Alpha 2
CHGA: Chromogranin-A
CI: Confidence intervals
CRNN: Cornulin
CTRC: Chymotrypsin-C
CVD: Cardiovascular disease
EFHD1: EF-hand domain-containing protein D1
ENPP5: Ectonucleotide pyrophosphatase/phosphodiesterase family member 5
eGFR: Estimated glomerular filtration rate
GAST: Gastrin
GWAS: Genome-wide association study
HbA_1c_: Glycated hemoglobin
HDL-C: High-density lipoprotein cholesterol
HR: Hazard ratio
HS3ST3B1: Heparan sulfate glucosamine 3-O-sulfotransferase 3B1
IDI: Integrated discrimination index
LASSO: Least absolute shrinkage and selection operator
MACE: Major adverse cardiovascular events
NHS: National health service
NMR: Nuclear magnetic resonance
NRI: Net reclassification index
NT-proBNP: N-terminal prohormone of brain natriuretic peptide
POSTN: Periostin
PRS: Polygenic risk score
ROC: Receiver operating characteristic
SBP: Systolic blood pressure
SD: Standard deviation
SMOC1: SPARC-related modular calcium-binding protein 1
T2D: Type 2 diabetes
TC: Total cholesterol
TNC: Tenascin-C
UKB: UK Biobank
WFDC2: WAP four-disulfide core domain protein 2
