# Supplementary material for: Improved sex-specific cardiovascular risk prediction with multi-omics data in people with type 2 diabetes
Source: Cardiovasc Diabetol. 2025 Dec 24;25:25. doi: 10.1186/s12933-025-03036-5 (PMC12837037; doi:10.1186/s12933-025-03036-5)
Supplement: Supplementary file 1 — Supplementary Material 1 [file 12933_2025_3036_MOESM1_ESM.docx]

**Supplemental Materials to**

**Improved sex-specific cardiovascular risk prediction with multi-omics data in people with type 2 diabetes**

Table of Contents

[Supplemental Table S1. Selected multi-omic biomarkers and data sources 2](#_Toc31994)

[Supplemental Table S2. Definition of endpoint major cardiovascular event (MACE) 3](#_Toc3681)

[Supplemental Table S3. ß-coefficients of the variables of the SCORE2-Diabetes model extended by 15 proteins for 10-year prediction of major cardiovascular events 4](#_Toc7752)

[Supplemental Table S4. NRI and IDI for 10-year MACE prediction by extending the SCORE2-Diabetes model with single- and multi-omics data (N=990) 6](#_Toc25393)

[Supplemental Table S5. ß-coefficients of the variables of the SCORE2-Diabetes model extended by selected multi-omics biomarkers for 10-year prediction of major cardiovascular events 7](#_Toc13259)

[Supplemental Figure S1. Flow charts of participant selection for proteomics-based and multi-omics-based cardiovascular risk prediction analyses 9](#_Toc11368)

[Supplemental Figure S2. ROC curves comparing the discrimination of the SCORE2-Diabetes model with and without proteomics extension for 10-year MACE risk (N=1,751) 10](#_Toc27141)

[Supplemental Figure S3. Calibration curves of the SCORE2-Diabetes model with and without proteomics extension for 10-year MACE risk prediction (N=1,751) 11](#_Toc18119)

[Supplemental Figure S4. ROC curves comparing the discrimination of the SCORE2-Diabetes model with and without multi-omics extension for 10-year MACE risk (N=990) 12](#_Toc3045)

[Supplemental Figure S5. Calibration curves of the SCORE2-Diabetes model with and without multi-omics extension for 10-year MACE risk prediction (N=990) 13](#_Toc5582)

[Supplemental Figure S6. Associations of selected multi-omics biomarkers and 10-year risk of MACE (N=990) 14](#_Toc27128)

[Supplemental Figure S7. Spearman correlation matrices for selected multi-omics biomarkers (N=990) 15](#_Toc24250)

**Supplemental Table S1.** Selected multi-omic biomarkers and data sources

| **Omics** | **Males** | **Females** | **Reference** |
| --- | --- | --- | --- |
| Genes | PRS for CVD with 1.42 million non-zero SNP variants | PRS for CVD with 1.42 million non-zero SNP variants | The CVD-PRS was derived using a genome-wide meta-analysis of 9 external GWAS datasets comprising 1,840,289 individuals and has been previously published [21]. |
| Metabolites | Acetate, albumin, creatinine, omega-3-pct, VLDL-size | Albumin, creatinine, GlycA, lactate | The metabolites were selected using sex-specific LASSO regression in the UKB (n=7,180). See previous work [22]. |
| Proteins | ACTA2, CHGA, CRNN, ENPP5, GAST, NT-proBNP, POSTN, TRIM21, WFDC2 | CTRC, EFHD1, HS3ST3B1, IL22, SMOC1, TNC, WFDC2 | The proteins were selected using sex-specific LASSO regression in this study using UKB data (n=1,751). |

**Abbreviations:** ACTA2, Actin Alpha 2; CHGA, Chromogranin-A; CRNN, Cornulin; CTRC, Chymotrypsin-C; CVD, Cardiovascular Disease; EFHD1, EF-hand domain-containing protein D1; ENPP5, Ectonucleotide pyrophosphatase/phosphodiesterase family member 5; GAST, Gastrin; HS3ST3B1, Heparan sulfate glucosamine 3-O-sulfotransferase 3B1; IL22, Interleukin-22; NT-proBNP, N-terminal prohormone of brain natriuretic peptide; Omega-3-pct, Omega-3 fatty acids percentage of total fatty acids; PRS, polygenic risk score; SMOC1, SPARC-related modular calcium-binding protein 1; SNP, single nucleotide polymorphism; TNC, Tenascin-C; TRIM21; E3 ubiquitin-protein ligase TRIM21; VLDL-size, Average diameter for very-low-density lipoprotein particles; WFDC2, WAP four-disulfide core domain protein.

**Supplemental Table S2.** Definition of endpoint major cardiovascular event (MACE)

| **Fatal MACE – cause-specific mortality due to any of the following:** | |
| --- | --- |
| *Endpoints included* | *ICD10-codes* |
| Hypertensive disease | I10-16 |
| Ischemic heart disease | I20-25 |
| Arrhythmias, heart failure | I46-52 |
| Cerebrovascular disease | I60-69 |
| Atherosclerosis/aortic aneurysm | I70-73 |
| Sudden death and death within 24 hours of symptom onset | R96.0-96.1 |
|  |  |
| *Endpoints excluded from the above endpoint:* | *ICD10-codes* |
| Myocarditis, unspecified | I51.4 |
| Subarachnoid haemorrhage | I60 |
| Subdural hemorrhage | I62 |
| Cerebral aneurysm | I67.1 |
| Cerebral arteritis | I68.2 |
| Moyamoya | I67.5 |
|  |  |
| **Non-fatal MACE** | *ICD10-codes* |
| Non-fatal myocardial infarction | I21-I23 |
| Non-fatal stroke | I61, I63-I66, I69 |

# **Supplemental** **Table S3.** ß-coefficients of the variables of the SCORE2-Diabetes model extended by 15 proteins for 10-year prediction of major cardiovascular events

| **Risk factor (units)** | **Coefficient** | |
| --- | --- | --- |
|  | **Male** | **Female** |
| **SCORE2-Diabetes variables** |  |  |
| Age (per 5 years) | -0.0897 | 0.6155 |
| Current smoking | -0.1200 | 0.0700 |
| SBP (per 20 mmHg) | -0.0720 | 0.0787 |
| Total cholesterol (per 1 mmol/L) | 0.2928 | 0.1959 |
| HDL cholesterol (per 0.5 mmol/L) | -0.0420 | -0.2396 |
| Smoking interaction with age | -0.0378 | -0.0678 |
| SBP interaction with age | 0.1047 | -0.1130 |
| Total cholesterol interaction with age | -0.0626 | -0.1599 |
| HDL interaction with age | 0.0193 | 0.2325 |
| Diabetes age at diagnosis (per 5 years) | 0.0113 | -0.0997 |
| HbA_1c_ (per 9.34 mmol/mol) | 0.1009 | -0.0455 |
| Ln eGFR (per 0.15 ml/min/1.73m^2^) | 0.2262 | 0.1099 |
| Ln eGFR^2^ (quadratic term) | 0.0101 | 0.0137 |
| HbA_1c_ interaction with age | 0.0192 | -0.1586 |
| Ln eGFR interaction with age | 0.0197 | 0.0691 |
| **Additional proteins** **(per 1 SD)** |  |  |
| ACTA2 | 0.3303 | - |
| CHGA | 0.1079 | - |
| CRNN | -0.2856 | - |
| CTRC | - | -0.7099 |
| EFHD1 | - | 0.5865 |
| ENPP5 | -0.3111 | - |
| GAST | 0.0965 | - |
| HS3ST3B1 | - | 0.4250 |
| IL22 | - | 0.4918 |
| NT-proBNP | 0.2030 | - |
| POSTN | 0.3851 | - |
| SMOC1 | - | 0.4752 |
| TNC | - | 0.6270 |
| TRIM21 | -0.3095 | - |
| WFDC2 | 0.8349 | 0.4915 |

**Abbreviations:** ACTA2, Actin Alpha 2; CHGA, Chromogranin-A; CRNN, Cornulin; CTRC, Chymotrypsin-C; EFHD1, EF-hand domain-containing protein D1; eGFR, estimated glomerular filtration rate; ENPP5, Ectonucleotide pyrophosphatase/phosphodiesterase family member 5; GAST, Gastrin; HbA_1c_, glycated hemoglobin; HDL, High-density lipoprotein; HS3ST3B1, Heparan sulfate glucosamine 3-O-sulfotransferase 3B1; IL22, Interleukin-22; NT-proBNP, N-terminal prohormone of brain natriuretic peptide; POSTN, Periostin; SBP, systolic blood pressure; SD, standard deviation; SMOC1, SPARC-related modular calcium-binding protein 1; TNC, Tenascin-C; TRIM21; E3 ubiquitin-protein ligase TRIM21; WFDC2, WAP four-disulfide core domain protein.

**Supplemental Table S4.** NRI and IDI for 10-year MACE prediction by extending the SCORE2-Diabetes model with single- and multi-omics data (N=990)

|  | **NRI** | | | | | |  | **IDI** | | | | | | |
| --- | --- | --- | --- | --- | --- | --- | --- | --- | --- | --- | --- | --- | --- | --- |
|  | **Total** | | **Male** | | **Female** | |  | **Total** | | **Male** | | **Female** | | |
|  | **NRI_total(%)_ (95% CI)** | **P-value^a^** | **NRI_total(%)_ (95% CI)** | **P-value^a^** | **NRI_total(%)_ (95% CI)** | **P-value^a^** |  | **IDI (95%CI)** | **P-value^a^** | **IDI (95% CI)** | **P-value^a^** | **IDI (95% CI)** | **P-value^a^** | |
| **Panel A** | | | | | | | | | | | | | |  |
| **SCORE2-Diabetes (ref)** |  | | | | | |  |  | | | | | | |
| **SCORE2-Diabetes+PRS** | **2.6  (-4.1, 11.8)** | **0.526** | **15.5  (-3.0, 32.9)** | **0.090** | **6.0  (-4.2, 11.4)** | **0.130** |  | **0.015 (0.003, 0.029)** | **0.040** | **0.019 (0.001, 0.035)** | **0.040** | **0.002  (-0.021, 0.020)** | **0.832** | |
| **SCORE2-Diabetes+Metabolomics** | **3.8  (-8.7, 11.4)** | **0.462** | **7.7  (-10.5, 16.2)** | **0.258** | **4.1  (-8.0, 16.1)** | **0.502** |  | **0.003  (-0.016, 0.027)** | **0.812** | **0.005  (-0.019, 0.033)** | **0.535** | **0.006  (-0.028, 0.040)** | **0.693** | |
| **SCORE2-Diabetes+Proteomics** | **30.6  (23.8, 47.8)** | **<0.001** | **51.2  (18.3, 67.9)** | **<0.001** | **29.5  (18.4, 42.1)** | **<0.001** |  | **0.098 (0.052, 0.130)** | **<0.001** | **0.105 (0.058, 0.146)** | **<0.001** | **0.111 (0.028, 0.210** | **<0.001** | |
| **Panel B** | | | | | | | | | | | | | |  |
| **SCORE2-Diabetes+Proteomics (ref)** |  | | | | | |  |  | | | | | | |
| **SCORE2-Diabetes+Proteomics+PRS** | **3.1  (-4.5, 3.3)** | **0.948** | **-1.5  (-7.0, 4.1)** | **0.595** | **1.9  (-6.3, 5.9)** | **0.538** |  | **-0.003  (-0.010, 0.003)** | **0.535** | **-0.005  (-0.017, 0.003)** | **0.238** | **0.000  (-0.006, 0.006)** | **0.931** | |
| **SCORE2-Diabetes+Proteomics+Metabolomics** | **-0.8  (-5.3, 7.7)** | **0.811** | **9.8  (-5.4, 17.0)** | **0.086** | **1.9  (-9.5, 12.4)** | **0.733** |  | **0.001  (-0.023, 0.027)** | **0.812** | **-0.005  (-0.037, 0.027)** | **0.792** | **-0.002  (-0.022, 0.028)** | **0.832** | |
| **Panel C** | | | | | | | | | | | | | |  |
| **SCORE2-Diabetes+Proteomics+Metabolomics (ref)** |  | | | | | |  |  | | | | | | |
| **SCORE2-Diabetes+Multi-omics** | **-1.4  (-3.7, 1.9)** | **0.339** | **-2.6  (-4.4, 3.9)** | **0.220** | **0.3  (-4.6, 4.4)** | **0.895** |  | **-0.002  (-0.005, 0.002)** | **0.455** | **-0.002  (-0.009, 0.003)** | **0.495** | **-0.001  (-0.006, 0.003)** | **0.752** | |

^a^*P*-values indicate the statistical significance of the change in NRI or IDI compared to the reference (ref) model in each respective panel. The NRI was calculated based on predefined 10-year MACE risk categories: 0–15%, >15–30%, and >30%. Statistically significant values (*P* < 0.05) are shown in bold.

**Abbreviations:** CI, confidence interval; IDI, integrated discrimination index; MACE, major adverse cardiovascular event; NRI, net reclassification index; PRS, polygenic risk score.

# **Supplemental Table S5.** ß-coefficients of the variables of the SCORE2-Diabetes model extended by selected multi-omics biomarkers for 10-year prediction of major cardiovascular events

| **Risk factor (units)** | **Coefficient** | |
| --- | --- | --- |
|  | **Male** | **Female** |
| **SCORE2-Diabetes variables** |  |  |
| Age (per 5 years) | -0.6712 | 0.9063 |
| Current smoking | -0.3326 | -0.0181 |
| SBP (per 20 mmHg) | -0.2602 | 0.0900 |
| Total cholesterol (per 1 mmol/L) | 0.2097 | 0.2351 |
| HDL cholesterol (per 0.5 mmol/L) | 0.0064 | -0.5594 |
| Smoking interaction with age | -0.0378 | -0.0678 |
| SBP interaction with age | 0.2976 | -0.0918 |
| Total cholesterol interaction with age | -0.2730 | -0.0340 |
| HDL interaction with age | -0.0264 | 0.2162 |
| Diabetes age at diagnosis (per 5 years) | 0.0051 | -0.1638 |
| HbA_1c_ (per 9.34 mmol/mol) | 0.0470 | 0.0147 |
| Ln eGFR (per 0.15 ml/min/1.73m^2^) | -0.2007 | -0.1060 |
| Ln eGFR^2^ (quadratic term) | -0.0115 | 0.0291 |
| HbA_1c_ interaction with age | -0.0093 | -0.2434 |
| Ln eGFR interaction with age | 0.0085 | 0.0364 |
| **Additional PRS** **(per 1 SD)** |  |  |
| CVD-PRS | 0.0673 | 0.0403 |
| **Additional metabolites** **(per 1 SD)** |  |  |
| Acetate | -0.1210 | - |
| Albumin | 0.3158 | 0.1281 |
| Creatinine | -0.3306 | -0.3978 |
| GlycA | - | -0.0636 |
| Omega-3-pct | -0.1954 | - |
| Lactate | - | 0.1075 |
| VLDL-size | -0.0901 | - |
| **Additional proteins** **(per 1 SD)** |  |  |
| ACTA2 | 0.1979 | - |
| CHGA | 0.0220 | - |
| CRNN | -0.1997 | - |
| CTRC | - | -0.8490 |
| EFHD1 | - | 0.1114 |
| ENPP5 | -0.3899 | - |
| GAST | 0.2080 | - |
| HS3ST3B1 | - | 0.5037 |
| IL22 | - | 0.5579 |
| NT-proBNP | 0.1940 | - |
| POSTN | 0.4796 | - |
| SMOC1 | - | 0.2765 |
| TNC | - | 0.6748 |
| TRIM21 | -0.4930 | - |
| WFDC2 | 0.8517 | 0.5199 |

**Abbreviations:** ACTA2, Actin Alpha 2; CHGA, Chromogranin-A; CRNN, Cornulin; CTRC, Chymotrypsin-C; CVD, Cardiovascular Disease; EFHD1, EF-hand domain-containing protein D1; eGFR, estimated glomerular filtration rate; ENPP5, Ectonucleotide pyrophosphatase/phosphodiesterase family member 5; GAST, Gastrin; HbA_1c_, glycated hemoglobin; HDL, High-density lipoprotein; HS3ST3B1, Heparan sulfate glucosamine 3-O-sulfotransferase 3B1; IL22, Interleukin-22; NT-proBNP, N-terminal prohormone of brain natriuretic peptide; Omega-3-pct, The Omega-3 fatty acids percentage of total fatty acids; PRS, polygenic risk score; SBP, Systolic blood pressure; SD, standard deviation; SMOC1, SPARC-related modular calcium-binding protein 1; TNC, Tenascin-C; TRIM21; E3 ubiquitin-protein ligase TRIM21; VLDL-size, Average diameter for very-low-density lipoprotein particles; WFDC2, WAP four-disulfide core domain protein.

**
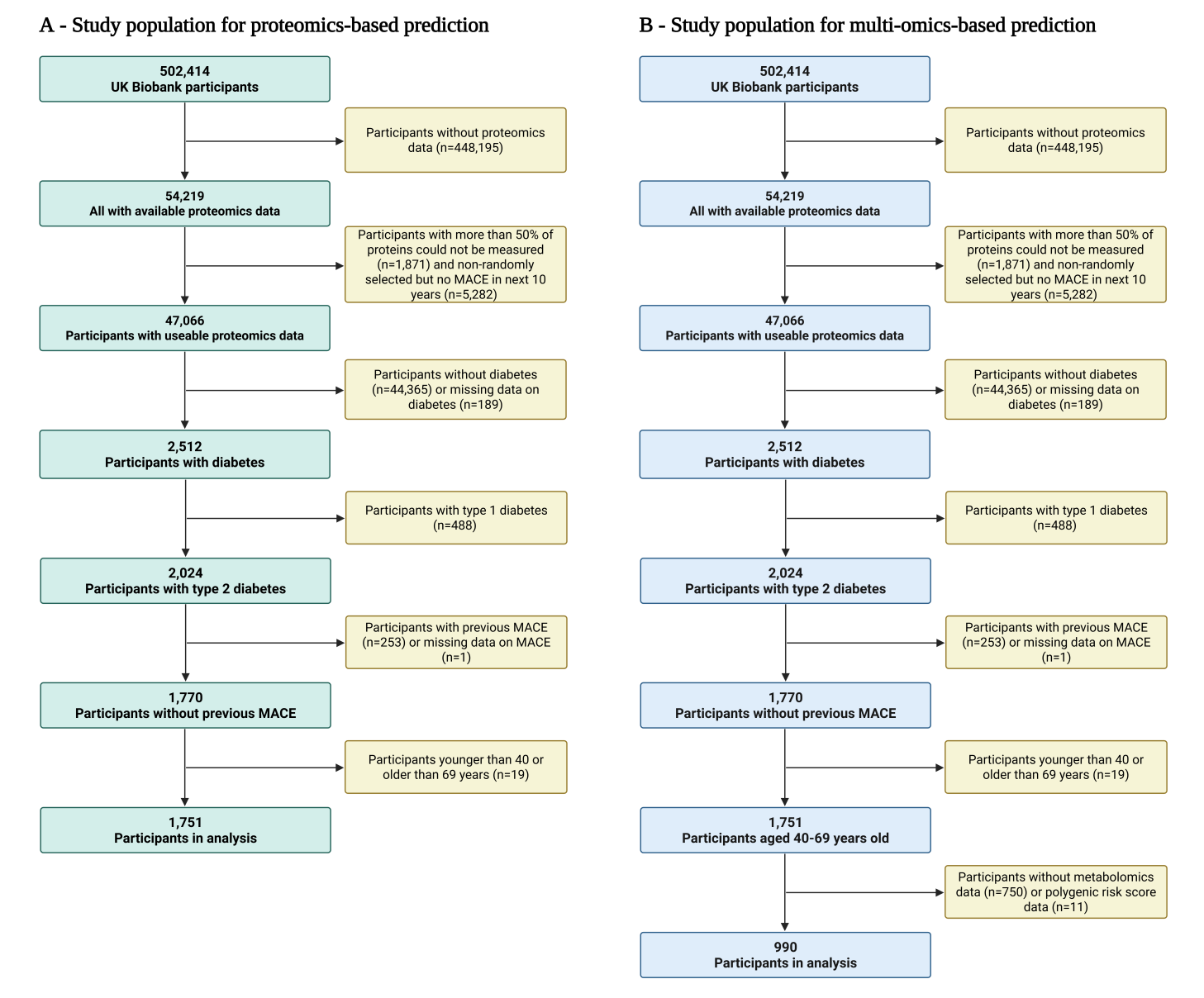
**

**Supplemental Figure S1. Flow charts of participant selection for proteomics-based and multi-omics-based cardiovascular risk prediction analyses**

**Panel A shows participant selection for the proteomics-based cohort; Panel B shows selection for the multi-omics cohort.**


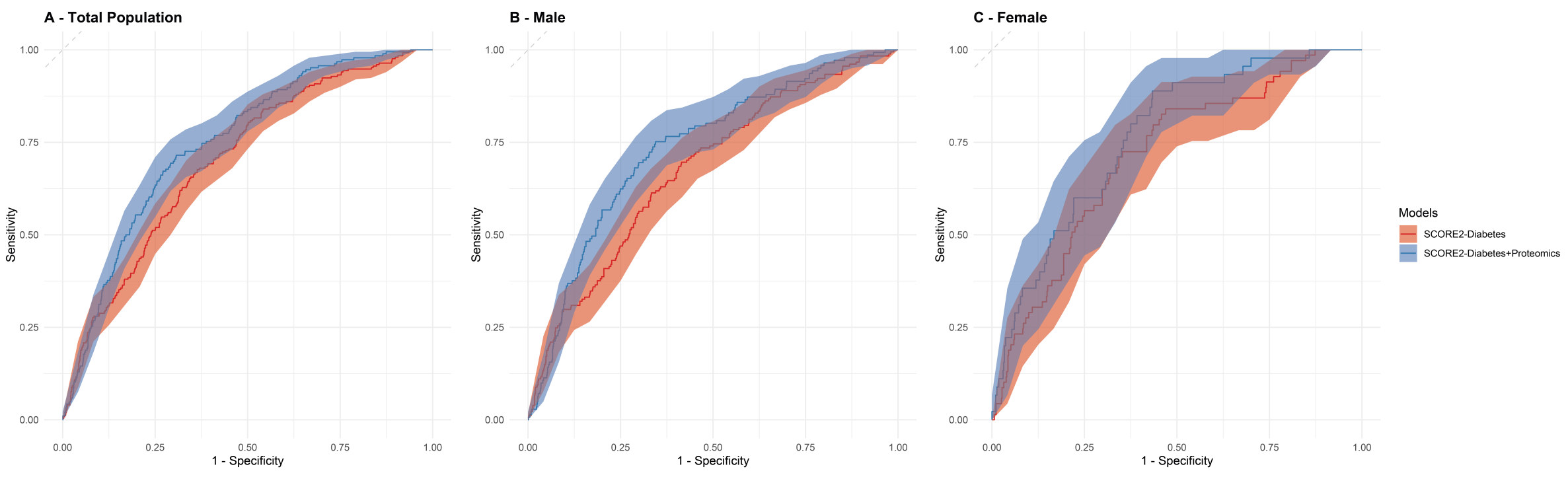


**Supplemental Figure S2. ROC curves comparing the discrimination of the SCORE2-Diabetes model with and without proteomics** extension **for 10-year MACE risk (N=1,751)**

Panels show receiver operating characteristic (ROC) curves for the SCORE2-Diabetes model (red) and the model extended with sex-specific proteomic signatures (blue) in the total population (**Panel A**), male participants (**Panel B**), and female participants (**Panel C**). Shaded areas represent 95% confidence intervals.

**Supplemental Figure S3.** Calibration curves of the SCORE2-Diabetes model with and without proteomics extension for 10-year MACE risk prediction (N=1,751)
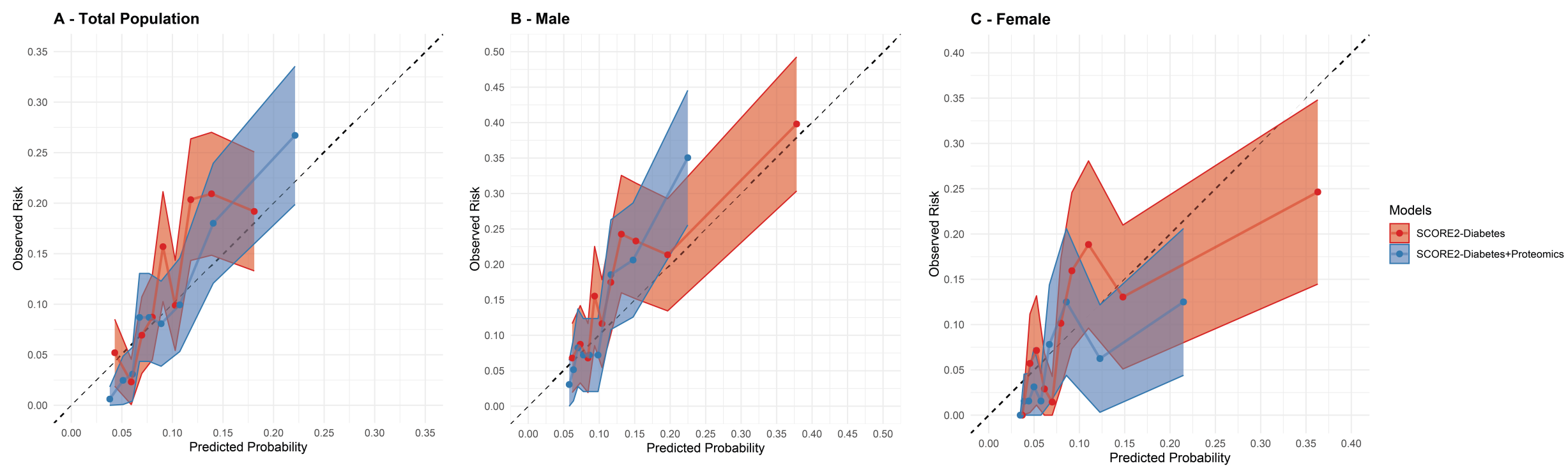


Calibration of the SCORE2-Diabetes model (red) and the extended model incorporating proteomic biomarkers (blue) is shown in the total population (**Panel A**), male participants (**Panel B**), and female participants (**Panel C**).


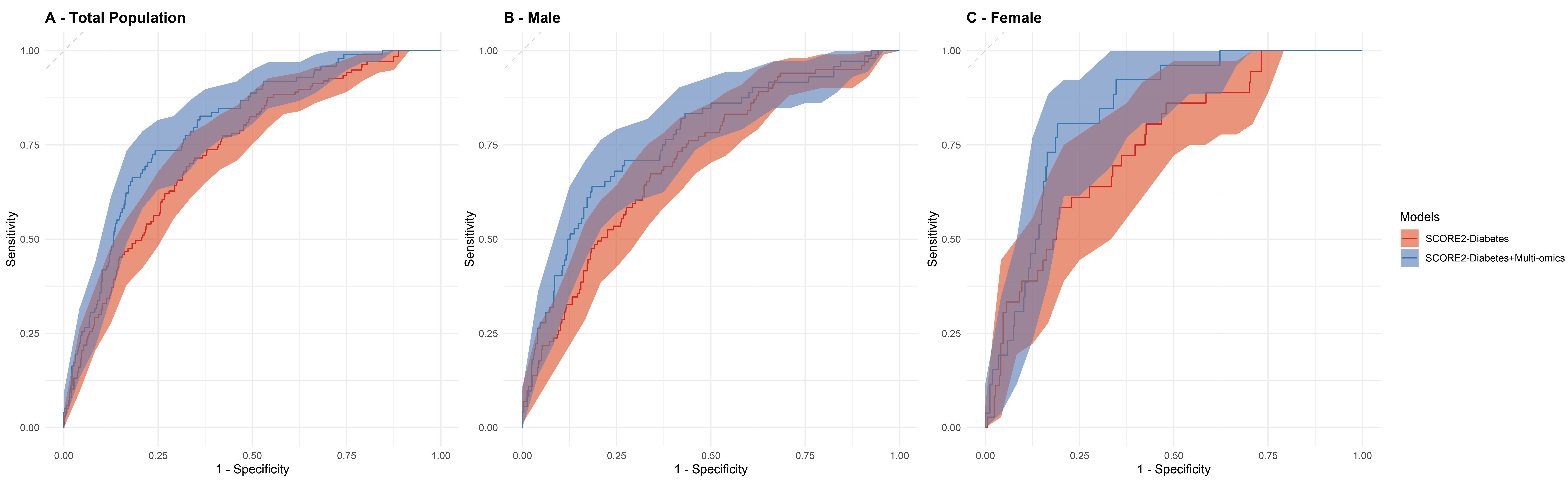


**Supplemental Figure S4. ROC curves comparing the discrimination of the SCORE2-Diabetes model with and without multi-omics** extension **for 10-year MACE risk (N=990)**

Panels display receiver operating characteristic (ROC) curves for the SCORE2-Diabetes model (red) and the model extended with multi-omics biomarkers (blue) in the total population (**Panel A**), male participants (**Panel B**), and female participants (**Panel C**). The multi-omics model refers to the integration of proteomics, metabolomics, and the CVD-PRS.

**
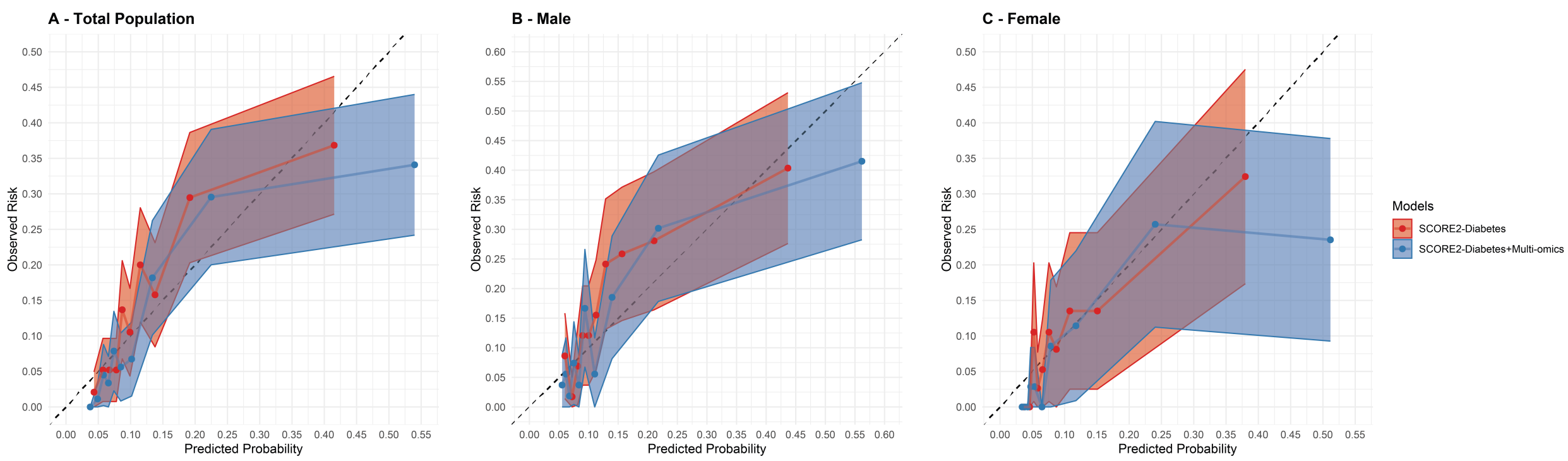
Supplemental Figure S5.** Calibration curves of the SCORE2-Diabetes model with and without multi-omics extension for 10-year MACE risk prediction (N=990)

Calibration of the SCORE2-Diabetes model (red) and the extended model incorporating multi-omic biomarkers (blue) is shown in the total population (**Panel A**), male participants (**Panel B**), and female participants (**Panel C**). The multi-omics model refers to the integration of proteomics, metabolomics, and the CVD-PRS.


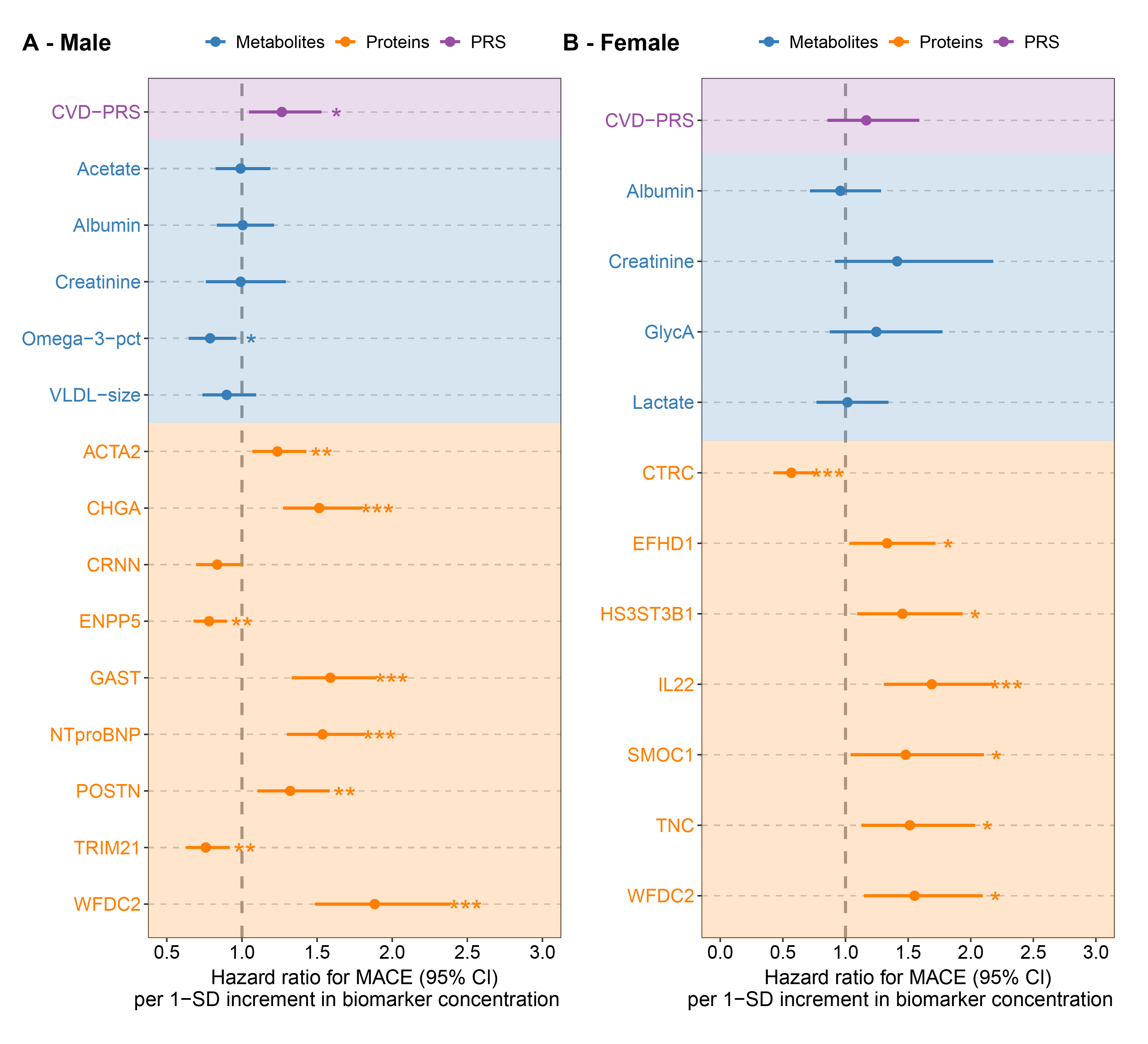


**Supplemental Figure S6. Associations of selected multi-omics biomarkers and 10-year risk of MACE (N=990)**

Forest plots display hazard ratios (HRs) and 95% confidence intervals (CIs) for incident MACE per 1-standard deviation (SD) increment in the concentration of each sex-specific biomarker. Analyses were conducted separately for male (**Panel A**) and female (**Panel B**) participants, with adjustments for all clinical covariates in the SCORE2-Diabetes model. Statistically significant associations are indicated by asterisks (* P < 0.05, ** P < 0.01, *** P < 0.001 assessed with FDR).

**Abbreviations:** HR, hazard ratio; CI, confidence interval; FDR, false discovery rate; other protein abbreviations as in **Figure S2**.


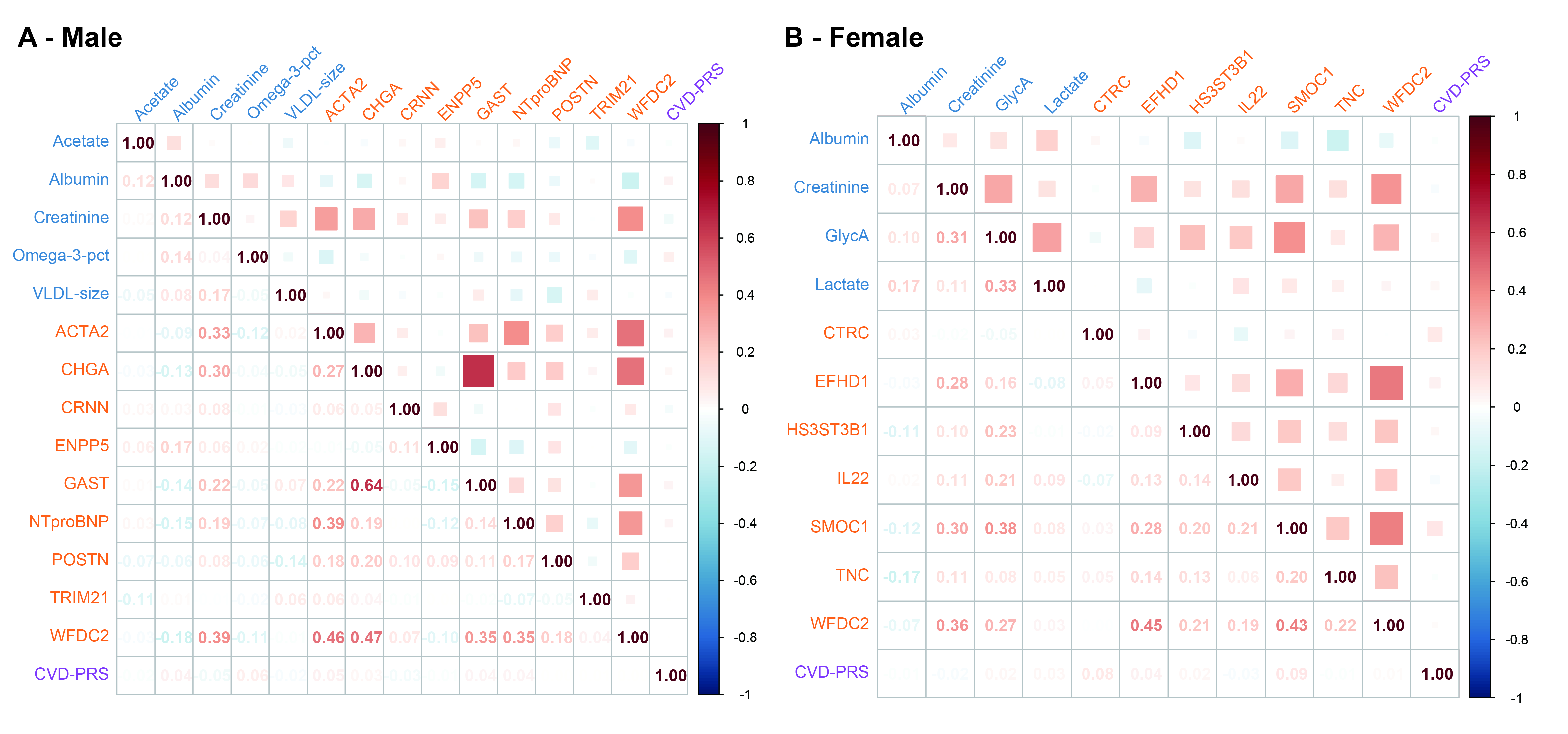


**Supplemental Figure S7. Spearman correlation matrices for selected multi-omics biomarkers (N=990)**

Correlation plots display the pairwise Spearman correlation coefficients among sex-specific multi-omics biomarkers in male (**Panel A**) and female (**Panel B**) participants

**Abbreviations:** ACTA2, Actin Alpha 2; CHGA, Chromogranin-A; CRNN, Cornulin; CTRC, Chymotrypsin-C; CVD, cardiovascular disease; EFHD1, EF-hand domain-containing protein D1; ENPP5, Ectonucleotide pyrophosphatase/phosphodiesterase family member 5; GAST, Gastrin; HS3ST3B1, Heparan sulfate glucosamine 3-O-sulfotransferase 3B1; IL22, Interleukin-22; NT-proBNP, N-terminal prohormone of brain natriuretic peptide; Omega-3-pct, The Omega-3 fatty acids percentage of total fatty acids; PRS, polygenic risk score; SMOC1, SPARC-related modular calcium-binding protein 1; TNC, Tenascin-C; TRIM21; E3 ubiquitin-protein ligase TRIM21; VLDL-size, Average diameter for very-low-density lipoprotein particles; WFDC2, WAP four-disulfide core domain protein.
